# Navigated Antral Bone Expansion (NABE): a prospective study on 35 patients with 4 months of follow-up post implant loading

**DOI:** 10.1186/s12903-020-01268-3

**Published:** 2020-10-07

**Authors:** Luigi V. Stefanelli, Nicola Pranno, Francesca De Angelis, Silvia La Rosa, Antonella Polimeni, Stefano Di Carlo

**Affiliations:** 1 Private practice, Prosthesis and Dental implant surgery, Rome, Italy; 2 Department of Oral and Maxillo-Facial Sciences, Sapienza University of Rome, 6. Caserta St., 00161 Rome, Italy; 3 Private Practice, Periodontics and Dental Implant Surgery, Tacoma, Washington, USA

**Keywords:** Dynamic navigation, Computer aided implantology, Sinus lift, Atrophic maxilla, Dynamic guidance

## Abstract

**Background:**

The insertion of dental implants in the atrophic posterior maxilla can be a challenge. One option is to modify the residual native bone in preparation for proper, prosthetically-driven implant placement. The procedure presented in this study is called Navigated Antral Bone Expansion (N.A.B.E). This procedure employs the use of a navigation system to plan and guide the initial pilot drilling, bone expansion, final site preparation, and implant insertion. The aim of this study was to compare the distance between the alveolar ridge and the sinus floor measured before and after the surgery performed using the N.A.B.E. technique.

**Methods:**

Thirty-seven partially edentulous patients who were candidates for implant supported restoration in the posterior maxilla, with a bone height ranging from 4 to 7 mm were enrolled. The N.A.B.E procedure was used to increase the bone height. Paired-samples t-test evaluated the distance between the alveolar ridge and the sinus floor measured before and after surgery. The occurrence of post-surgical complications, and the angular deviation between the planned osteotomy and the actual placed implant trajectories were evaluated.

**Results:**

Out of the 37 consecutive patients enrolled in the study, 35 were considered in the data analyses. Patients’ bone height after surgery compared to the bone height before surgery showed a statistically significant increase (*p* < .0005) of 3.96 mm (95% CI, 3.62 mm to 4.30 mm). No post-operative complications were observed in the 35 patients. The mean angular deviation between the planned osteotomy trajectory and the placed implant trajectory ranged between 12.70^0^ to 34.90^0^ (mean 25.17^0^ ± 5.10^0^).

**Conclusions:**

This study provides evidence that N.A.B.E. technique is able to provide a significant bone increase, and could be considered an alternative method to the management of the atrophic posterior maxilla with a minimally invasive approach.

## Background

The insertion of dental implants in the atrophic posterior maxilla can be a challenge due to a limited vertical alveolar bone height (often caused by post-extraction resorption of the alveolar bone or by a pneumatized sinus), low bone density (D3-D4) and difficult surgical access [1–4]. Several solutions are available for implant supported restorations in the atrophic posterior maxilla: sinus floor augmentation via lateral window approach or a trans-crestal augmentation, pterygoid or zygomatic implants, and short implants [5–16]. Bone stress distribution is influenced by bone quality, implant used (i.e. diameter, length, macrostructure), implant position, crown to implant length ratio, and type of the prosthetic restoration used. The effect of implant length on stress distribution seems to be noncritical except in D4 bone type, where the bone-implant interface length is an important key for success [17]. Another critical factor for success is the tilting degree of the implants [18]. This is important not only for reducing stress, but also for the achieving an ideal prosthetic position for the abutment-fixture (screw retained prosthesis is desired).

A pneumatized maxillary sinus may result in a deficient ridge height, inadequate for ideal restoratively-driven implant placement (prosthetic driven implantology), yet with adequate volume of residual bone in the vestibular, palatal, mesial or distal aspects, allowing for the placement of a tilted implant (bone guided implantology). Alternatively, the residual bone may be altered to allow for a more favorable implant position, that will better comply with the principles of prosthetically-driven implantology. The technique used is called Navigated Antral Bone Expansion (N.A.B.E). This technique can make an alternative for the use of short implants or for bone augmentation. The procedure consists of several steps: first – the initial osteotomy is prepared using a pilot drill, inserted in the available residual bone, although not in the ideal restoratively-driven direction; next - bone expanders are inserted into the osteotomy to further widen the bone; finally, the osteotomy’s direction is adjusted to the correct position. The use of a navigation system to plan and guide the initial pilot drilling, bone expansion, final site preparation and implant insertion may reduce the challenges and improve the predictability of the procedure. This technique is feasible when the residual bone is at least 4 mm wide. A major challenge in pursuing this approach is initiating the osteotomy with the pilot drill in the center of the narrow residual bone and angulating it so as not to perforate the cortical plates as it is inserted. The use of a navigation system [19–22] to plan and guide the initial pilot drilling, the final site preparation, and the implant insertion may reduce the challenge and improve the predictability of the procedure. In particular, the use of dynamic surgery allows the clinician to monitor each step and modify the plan as needed. The dynamic navigation system used in this study (Navident, ClaroNav, Canada) uses Trace Registration technology, which enables the clinician to use an existing CBCT (for the partially edentulous patients who have at least 3 remaining teeth), rather than preparing a custom stent and CT scanning the patient with a fiducial marker. Instead, registration of the jaw with its CT image is accomplished by tracing the existing teeth as natural fiducial markers [19, 20] and performing the guided surgery without any in-mouth appliance. This provides a full digital implant-prosthetic treatment workflow [23, 24].

The aim of the present study was to standardize a new method (N.A.B.E) as an alternative to short implants or bone augmentation, comparing the distance between the alveolar ridge and the sinus floor measured before the surgery using the N.A.B.E. technique and after it.

The technique’s success rate, the angular deviation between the planned osteotomy and the final implant position, as well as the type of custom-made prosthesis fixation system utilized (cemented or screw-retained) were evaluated.

## Methods

### Study design

This study was designed as an in-vivo, single blinded, prospective case series.

### Study population / demographics

Thirty-seven partially edentulous patients aged > 18 years in need of implant supported restoration in the posterior maxilla, with vertical and horizontal bone atrophy were recruited from the Department of Periodontics and Implant Dentistry at the Policlinico Umberto I, Sapienza University of Rome, Italy. Enrollment criteria were: (1) Patients with posterior maxillary atrophy, requiring restoration, with a residual bone height ranging from 4 to 7 mm (2) Patients with at least three stable / non-mobile teeth. (3) The presence of 10 mm or more of residual bone height and 4 mm or more of bone width in the palatal / vestibular or in the mesial / distal position relative to the ideal prosthetic plan. The following exclusion criteria were applied: (1) Patients with general contraindications to implant surgery (2) Patients with systemic diseases that could influence the outcome of the therapy (i.e. diabetes with glycated hemoglobin (HbA1c) ≥6.5%, osteoporosis or use of bisphosphonate medications.(3) Patients with a history of radiation to the head and neck region (4) Patients who are pregnant or nursing.

A written informed consent was obtained from each patient after a detailed description of the study protocol and treatment. The protocol was in accordance with the 1975 Declaration of Helsinki on medical protocols and ethics and its later amendments. The study protocol included the use of a post-operative CBCT scan to assess the position and angulation of the implants relative to the virtual plan. This study protocol was approved by the Department of Oral and Maxillofacial Sciences - Sapienza, University of Rome (Protocol identifying number: 582/17).

### Trace and Place (TaP) protocol

The TaP protocol consists of 3 steps. The first is the creation of a surgical plan using the information achieved from the DICOM (Digital Imaging Communication in Medicine) data acquired from a CBCT scan. The second is the selection of existing landmarks on the CBCT to record the patient’s jaw. The last step is the navigated implant placement according to the plan.

#### Plan

For each patient, a CBCT and an intraoral surface scan (IOS) of the treated jaw were obtained. An ideal virtual wax-up of the teeth to be replaced was done. The Navident software was used to superimpose the DICOM files from the CBCT and the STL files from the IOS, the overlapping was semi-automatically done using the residual teeth.

**Fig. 1:**
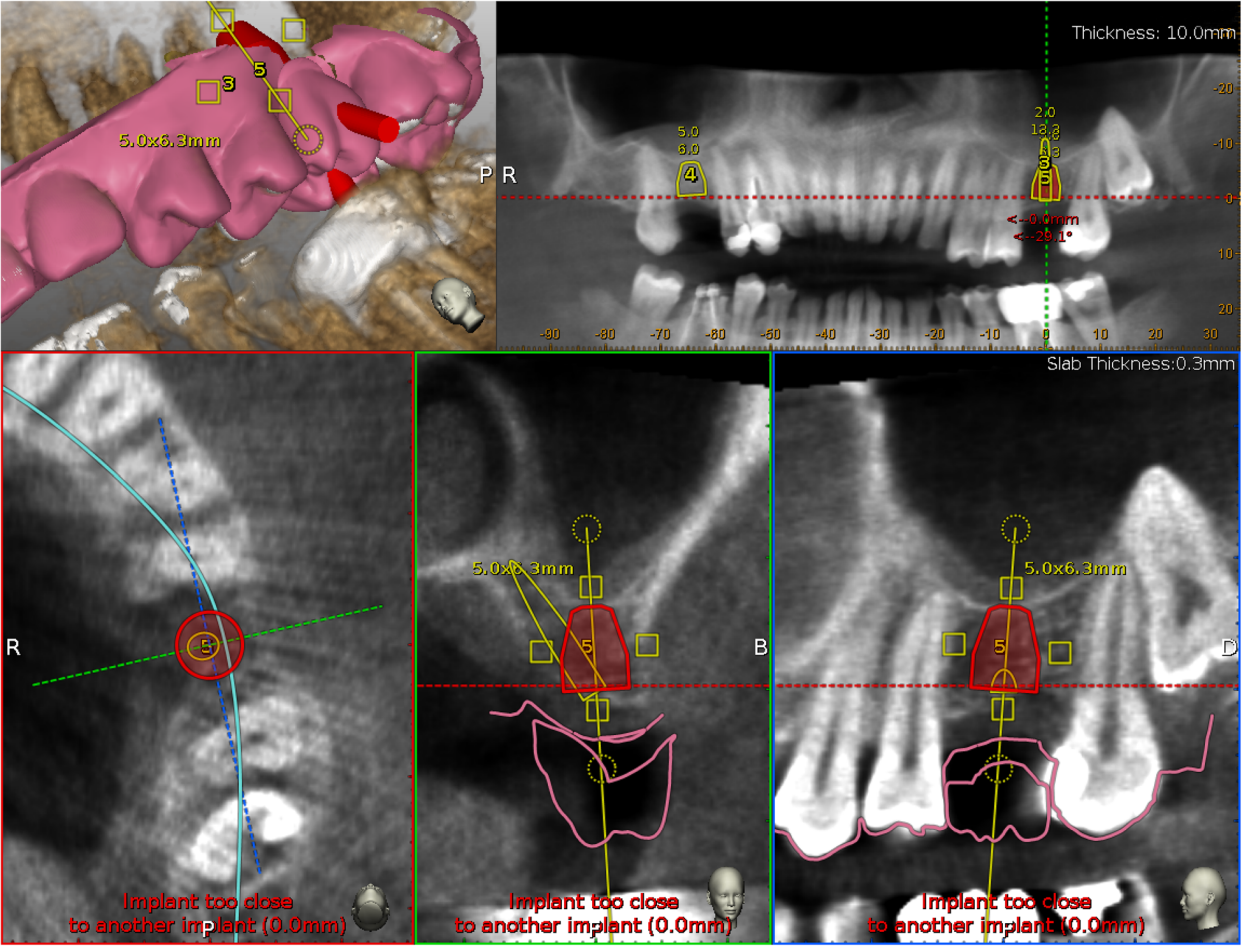
Implant planning using a STL file as reference for a prosthetic driven implant placement

Implant placement was then prosthetically planned utilizing the digital wax up of the missing tooth (Fig. 1). As part of the planning, the surgeon selected 3 to 6 landmarks on hard tissue structures, typically teeth, to be used as the starting points for tracing.

#### Trace

**Fig. 2:**
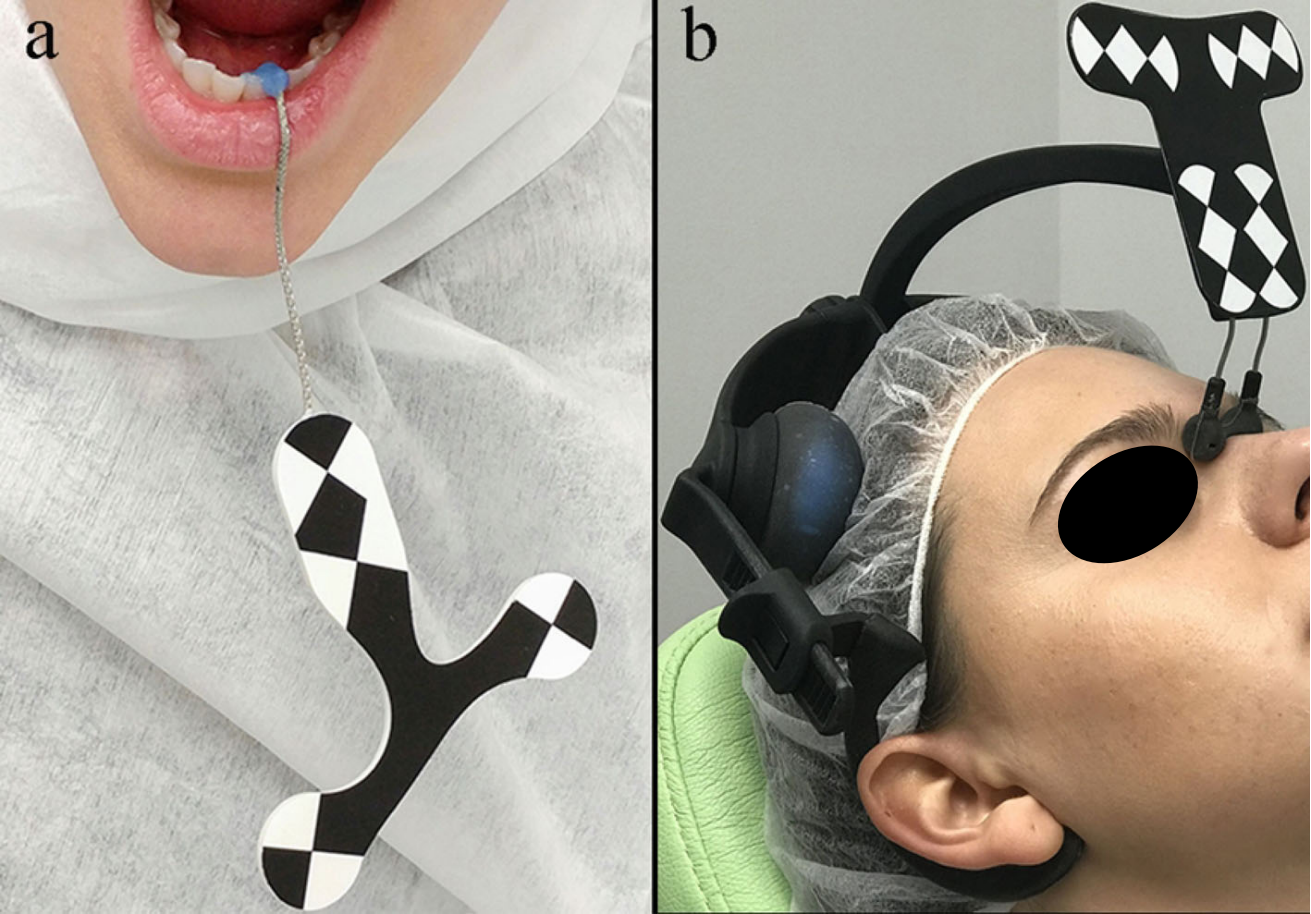
**a** Jaw tracker used in the lower jaw for dynamic navigation TaP. **b** Head tracker used in the upper jaw for dynamic navigation TaP

An optical tracking tag was fixed on the jaw on which surgery is being performed to track the patient’s jaw with the system’s camera. The jaw Tracker (consisting of an optical tag and bendable metal wire) must be connected to 1–2 teeth in the residual dentition with a light-cured composite resin (Fig. 2a). Alternatively, but only in the maxilla, a Head Tracker can be used for tracking of the maxilla by placing it directly on the patient’s head (Fig. 2b). After, a tracking tool was used starting at the landmark locations and sliding the tracer’s ball tip in full contact over the surface of each landmark until a 15 cm path has been traced.

**Fig. 3:**
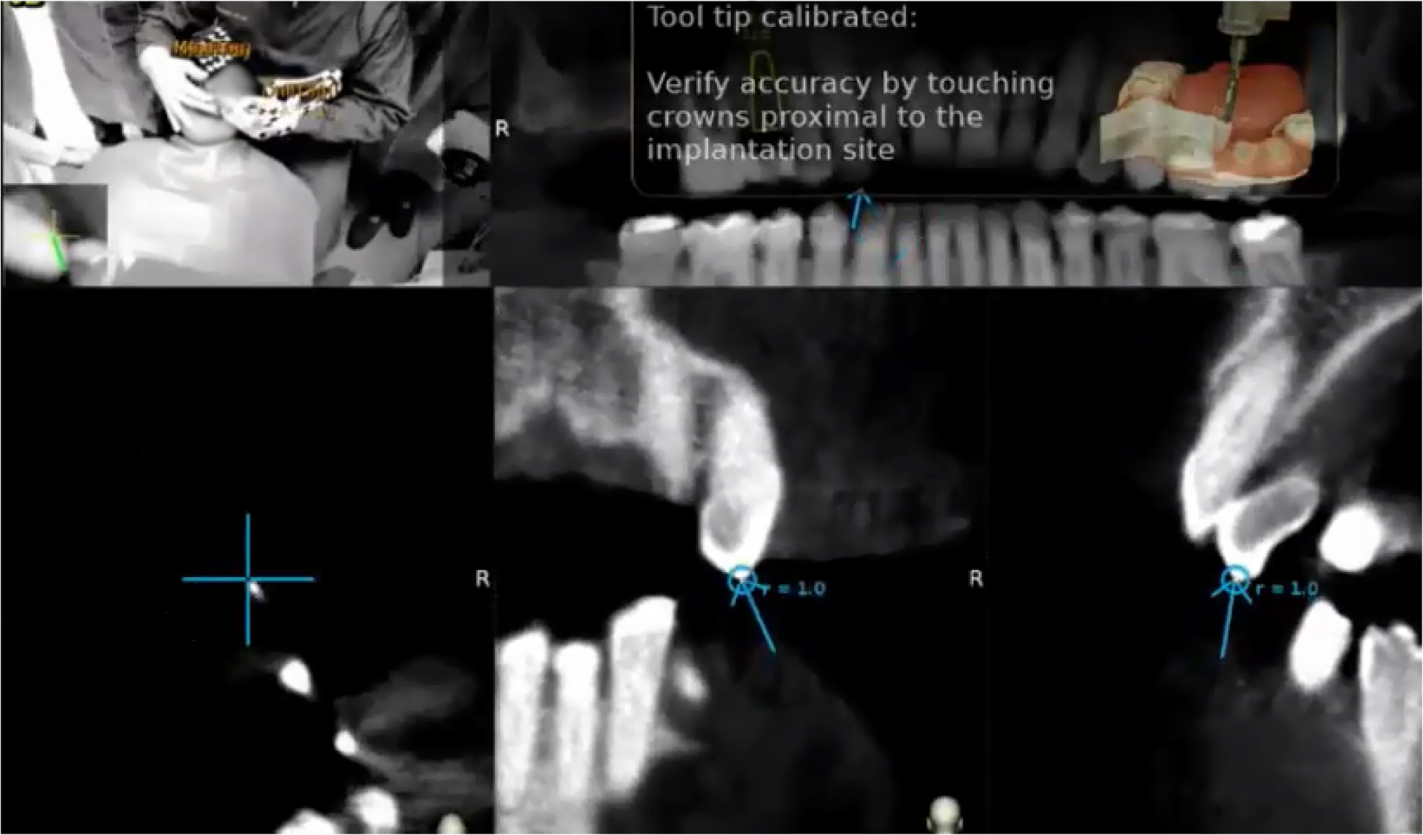
The surgeon can verify the registration accuracy by touching the tracer’s ball tip on the patient’s high contrast landmarks from several aspects and comparing the physical location of the tip with its on-screen representation on the system’s display

At the end of the registration, the software automatically performs the alignment between the trace points in the mouth of the patient and the same landmarks in the CBCT. The whole process is long approximately 1–2 min. The accuracy of the trace registration can be evaluated instantly by touching the tracer’s ball tip to the patient’s teeth from the buccal, lingual, incisal/occlusal, and proximal planes and comparing the actual physical location of the tracer tip with its on-screen representation on the system’s display (Fig. 3). The tracing steps can be repeated whether the registration accuracy obtained is not sufficient. The same process was used to calibrate the handpiece drill axis and tip.

#### Place

The navigated implant placement can be carried out following the target view that allows the clinician to verify, in real-time, the entry point, depth, and angulation of the osteotomy. The other views that the clinician can see on the screen are useful to follow the position of the drill in the coronal and sagittal views.

### Surgical treatment

All patients before the surgical treatment underwent an oral hygiene protocol consisting of polishing and supra- and subgingival debridement. In addition, patients received prophylactic antibiotic therapy with 2 g of Augmentin (GlaxoSmithKline, London, UK) One hour prior to surgery, Immediately before the procedure, and they were instructed to rinse with a 0.2% chlorhexidine digluconate solution (Corsodyl, GlaxoSmithKline Consumer Healthcare, Genval, Belgium) for 2 min. All surgical procedures were performed by the same experienced surgeon (L.V.S.). Local anesthesia with 2% mepivacaine 1:100,000 adrenalin (Carbocaine, AstraZeneca, Milan, Italy) was obtained, followed by buccal and lingual full-thickness flaps.

The N.A.B.E consists of seven steps: Pilot osteotomy, bone expansion, implant osteotomy for the planned implant site to the planned length, final bone expansion and axis correction, final implant site preparation and implant insertion.

**Fig. 4:**
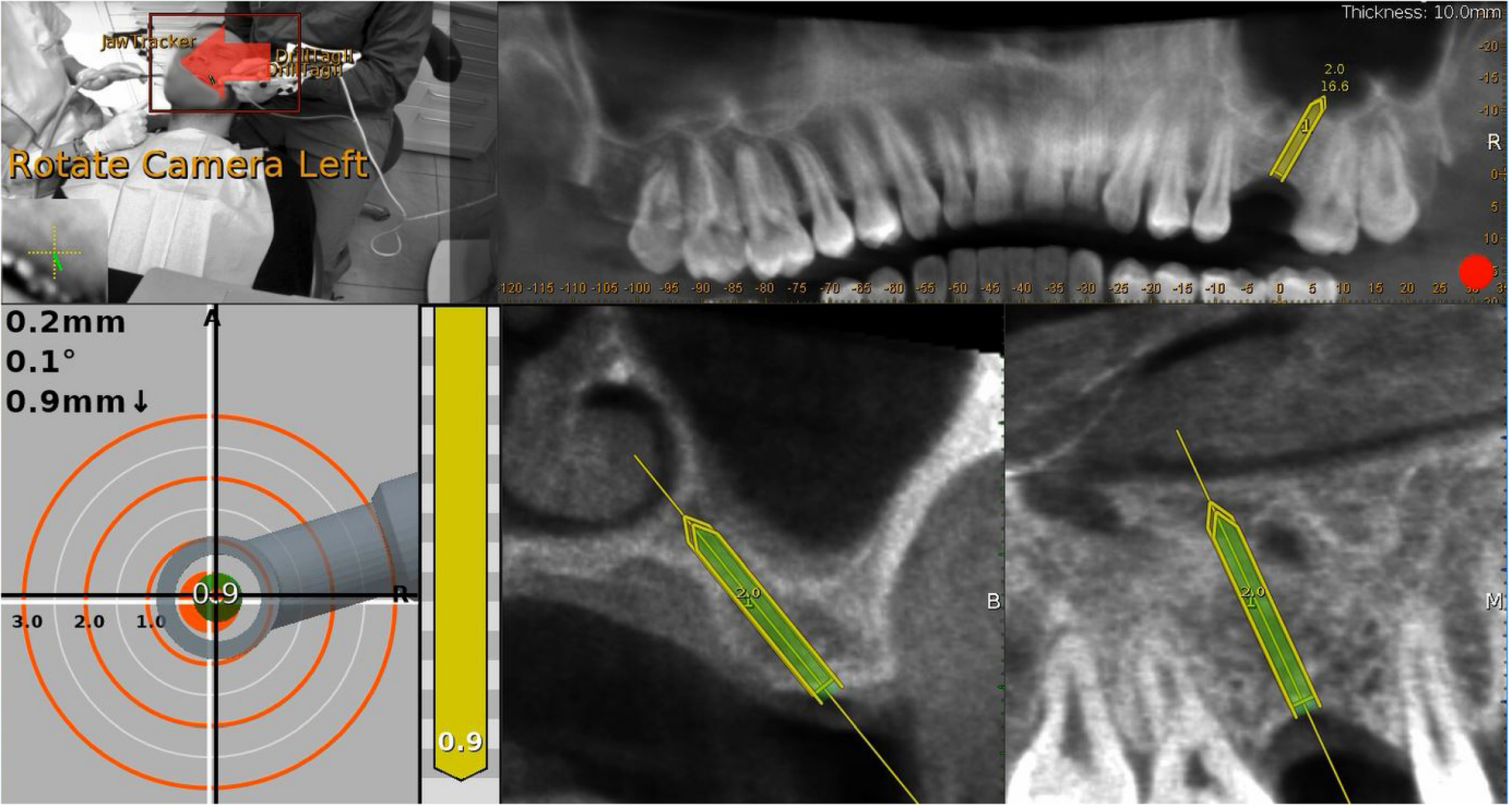
In the first step, the pilot drill was used to make the initial osteotomy using the available residual vertical bone height in the palatal and distal position relative to the restoratively driven implant planning position

**Fig. 5:**
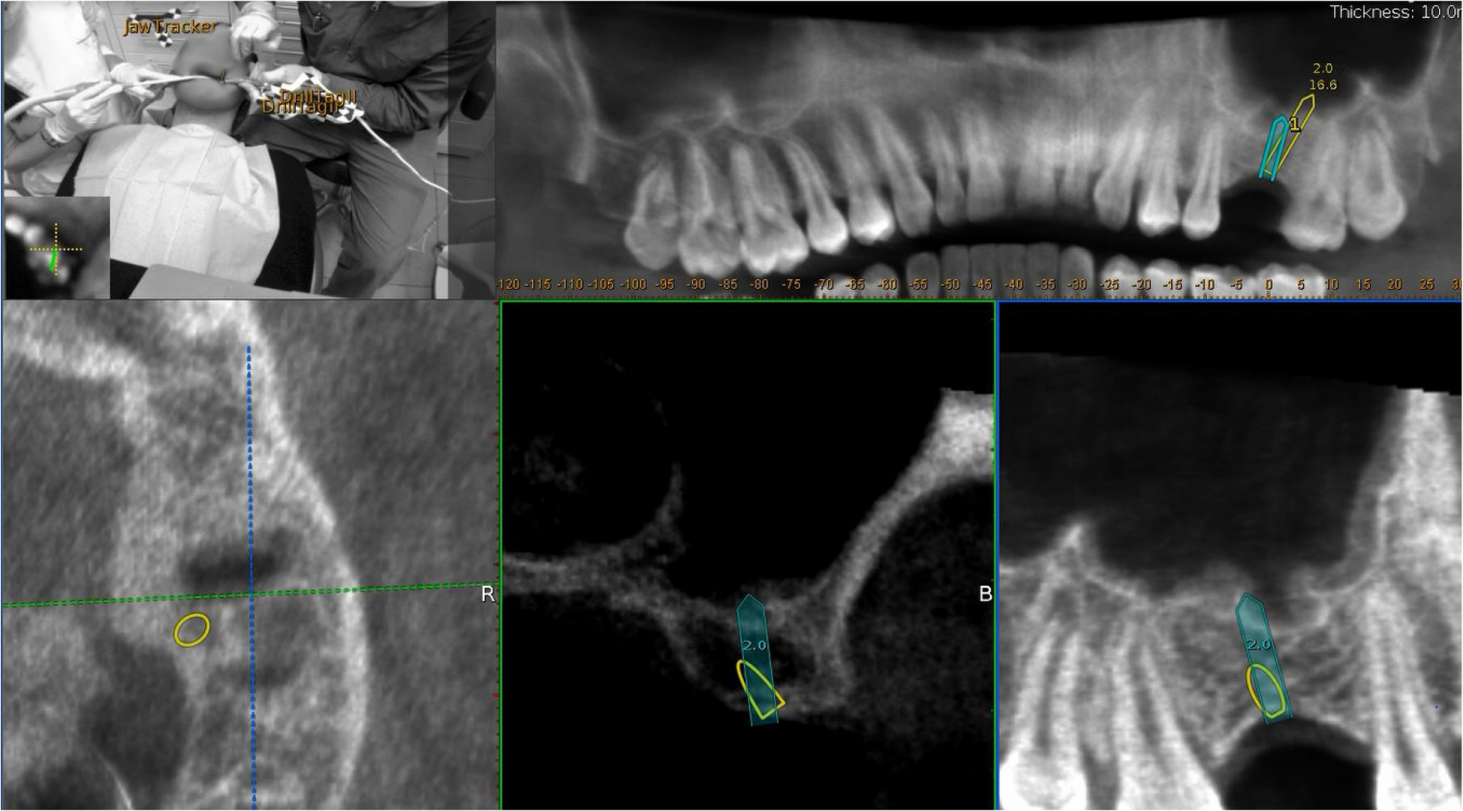
The bone expander is inserted in the same direction of the initial osteotomy when the last 3 mm of the working length is reached; the axis is corrected by using these last 3 mm

**Fig. 6:**
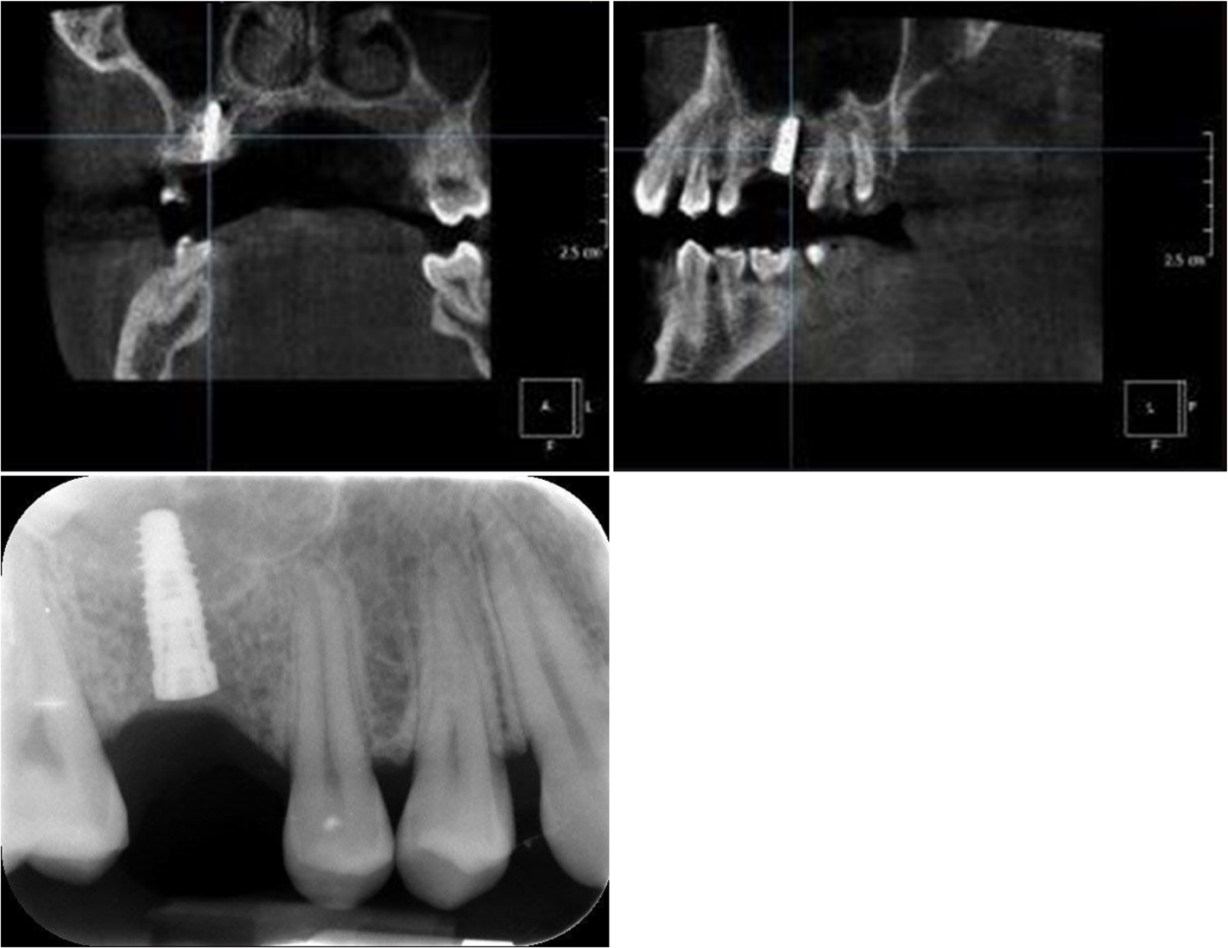
The final implant osteotomy in the correct position was performed and the implant inserted

In the first step, the pilot drill was used to make the initial osteotomy in the available residual bone height, either the palatal, vestibular, mesial or distal aspect relative to the ideal implant position (Fig. 4). Second, bone expansion was done in the same direction of the pilot drill. Since the sinus wall is the weakest and the bone expanders work only at the tip exerting lateral force, the expansion occured towards the sinus cavity. The bone expanders (Biohorizon tapered ridge expanders) were navigated, as the pilot drill, by the dynamic navigation system and with a low rotation speed (50 rounds/min). The first osteotomy of the ideal implant position is prepared to a length of 2 mm from the sinus floor. Using the bone expanders, it is possible to correct the axis to the implant plan. The bone expander was inserted in the same direction of the first pilot osteotomy and the first bone expanders, but when the last 3 mm of the working length was reached, the axis was corrected for the remaining 3 mm of insertion (Fig. 5). The drill sequence and implant placement were then completed, guided by Navident to follow the optimal placement plan, (Fig. 6). After implant insertion, the mucoperiosteal flaps were repositioned and stabilized without tension using resorbable interrupted sutures (5–0 Vicryl, Johnson & Johnson Medical, Norderstedt, Germany), which were removed after 2 weeks.

### Post-surgical protocol

The antibiotic protocol included administering 875 mg of amoxicillin plus 125 mg of clavulanic acid (Augmentin, GlaxoSmithKline, London, UK) twice daily for 7 days. After surgery, analgesia was achieved with 200 mg of ketoprofen (Ibifen, Aprilia, Latina, Italy) for a maximum of three times daily according to the needs of individual patients. In order to reduce the probability of complications, each patient was instructed to clean teeth with a soft toothbrush and to avoid the use of floss, interdental brushes, and toothpicks, and rinse 3 times daily with 0.12% chlorhexidine digluconate (Corsodyl, GlaxoSmithKline Consumer Healthcare) for 2 weeks, and to follow a soft diet for 1 week.

### Outcome measures

#### Radiographic assessment

The primary outcome was the result of the evaluation of bone height before and after N.A.B.E. Two independent investigators (F.D., N.P.) who were blinded to other measured the distance between the alveolar ridge and the sinus floor. Disagreement was solved by consensus, with a third investigator consulted when consensus could not be reached (defined as the difference between the measurements made by the two experts of > 0.1 mm).

#### Complications

The following post-surgery complications were recorded: (1) post-surgical complications associated with the N.A.B.E technique, (2) post-operative hemorrhage, (3) post-operative infection and (4) early implant failure.

#### Angle correction assessment

**Fig. 7:**
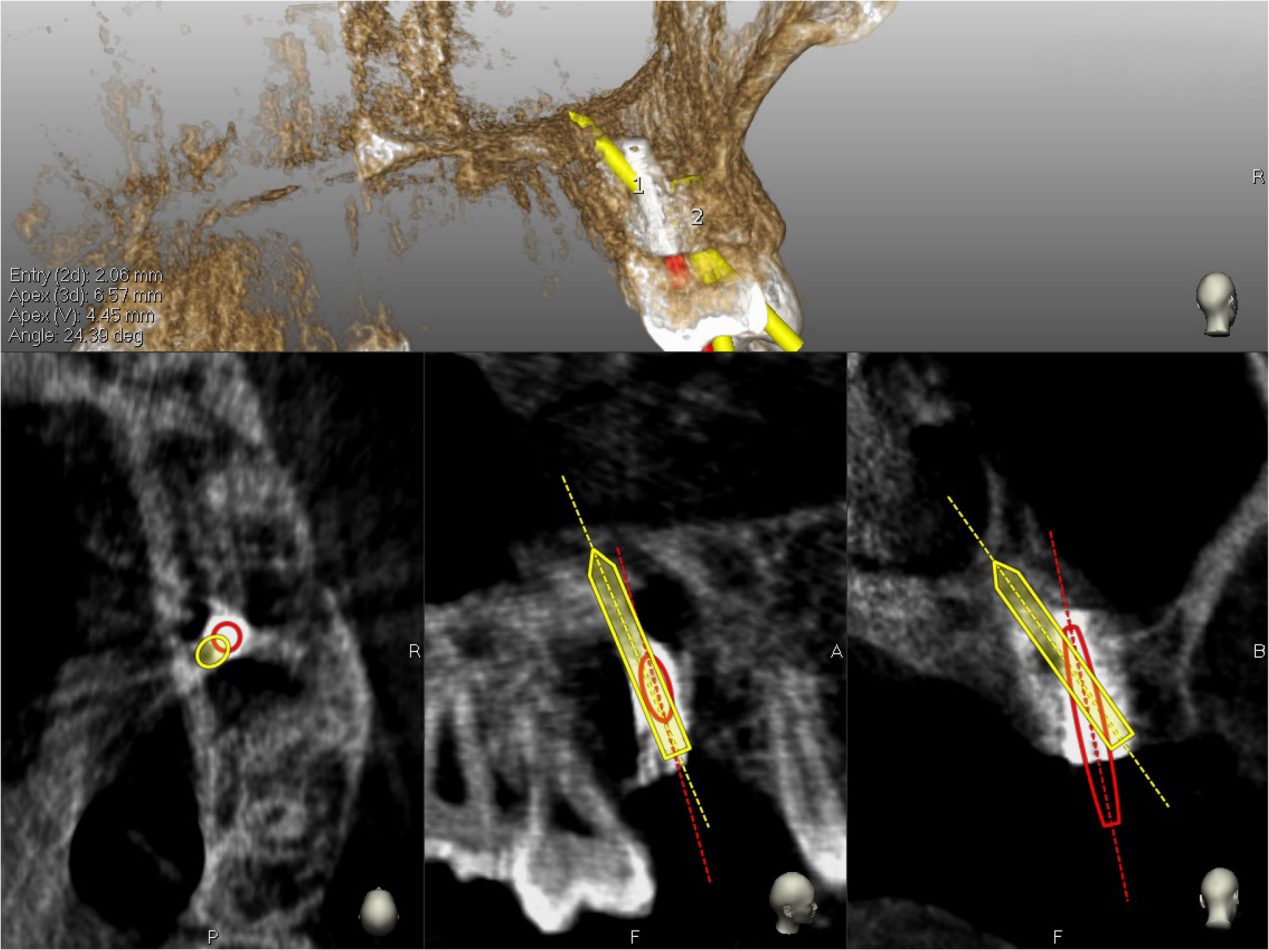
The software automatically fits a model of the implant to its appearance in the post-operative image and computes the angular axis corrected between the planned and actual implant locations

The preoperative surgical plan and the postoperative CBCT were superimposed using (EvaluNav), a software program provided as part of the Navident navigation system. The comparison was done directly between the two volumetric images. The software provides various visualization tools to confirm that the two images are precisely aligned. Once the user is satisfied with the volumetric registration, the software automatically fits a model of the implant to its appearance in the post-operative image and computes the spatial deviations between the planned and actual implant locations. EvaluNav is routinely used to calculate placement accuracy and in this case it was used to calculate the angular deviation from the pilot drill insertion to the final implant placement (Fig. 7).

### Statistical analysis

A database was created using Excel (Microsoft, Redmond, WA, USA). Descriptive statistics including mean ± SD values were calculated for each variable, and box plots were used to evaluate data outliers. The Shapiro-Wilk test was used to determine whether or not the data conformed to a normal distribution.

To identify if a statistically significant mean difference existed in the distance between the alveolar ridge and the sinus floor measured before and after surgery, a paired-samples t-test was used. Data were evaluated using standard statistical analysis software (SPSS version 20.0, Statistical Package for the Social Sciences, IBM Corporation, Armonk, NY, USA). In each test, the cut-off for statistical significance was *p* ≤ 0.05.

## Results

Out of the 37 consecutive patients enrolled in the study, 35 were considered in the data analysis. The two excluded subjects experienced a fracture of the palatal alveolar process, making primary implant stability impossible.

No post-operative complications were observed in the 35 remaining patients. All implants were osseointegrated after 4 months of healing. At the time of prosthetic loading an angled abutment was used in 3 out of 35 implants (8.6%) because the angle correction obtained with the N.A.B.E was insufficent for a straight abutment.

### Radiographic assessment

**Fig. 8:**
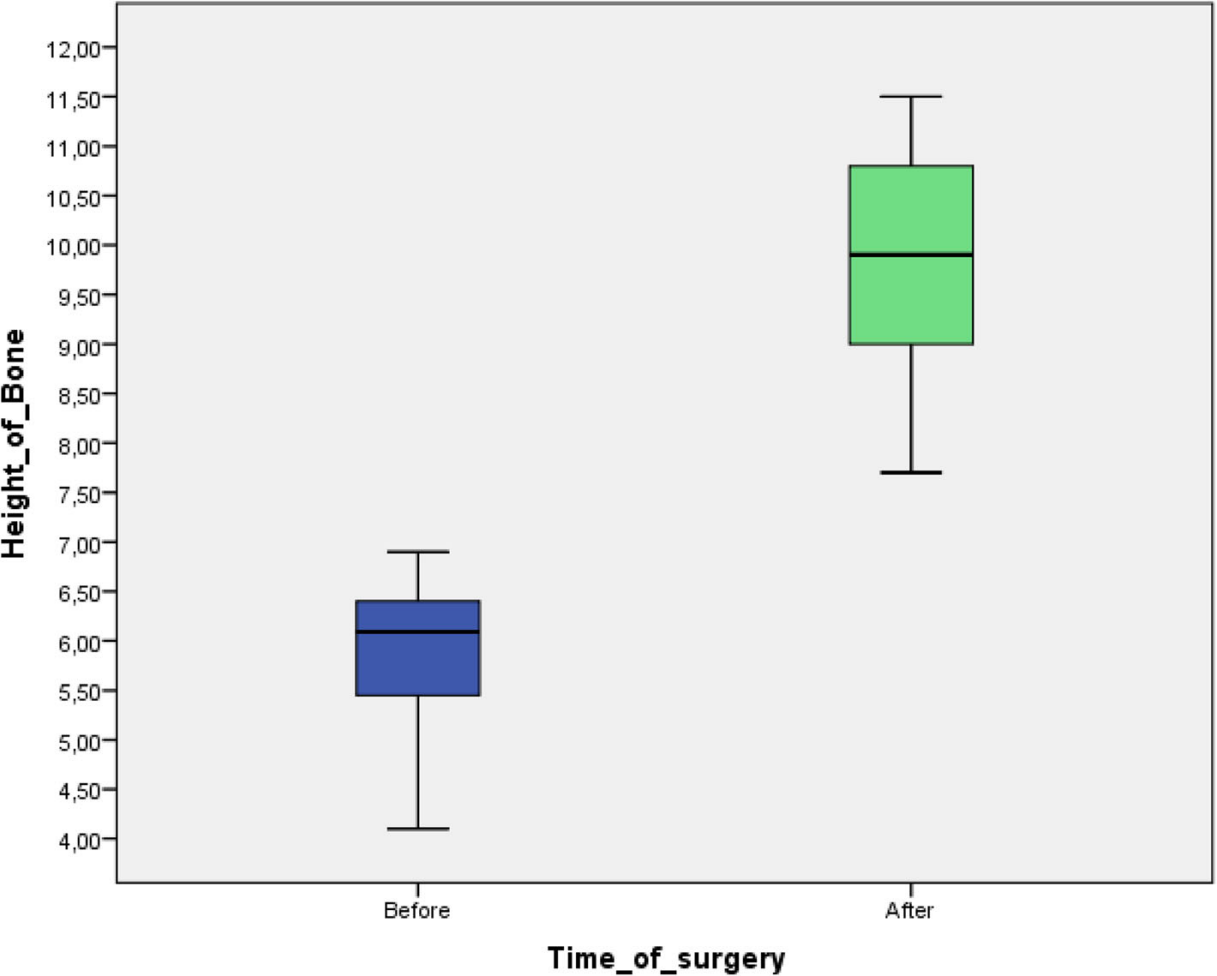
Box plots showing the median, quartile, and minimum and maximum values of the distance between the alveolar ridge and the sinus floor (mm) measured before and after surgery . Boxes contain 50% of all values; the horizontal lines inside the boxes indicate the medians and the vertical lines extend to 1.5 of the interquartile range

Patients included in the analysis presented with a residual bone height ranging from 4.10 to 6.90 mm (mean 5.89 ± 0.68 mm) demonstrated through linear measurements on CBCT cross-sectional slices. The bone height after the N.A.B.E ranged from 7.70 to 11.50 mm (mean 9.85 ± 1.10 mm) (Fig. 8). A paired-samples t-test was used to determine whether a statistically significant mean difference existed between the distance from the residual bone height at baseline compared to the bone height after the N.A.B.E. procedure. No outliers were detected. The assumption of normality was not violated, as assessed by the Shapiro-Wilk test (*p* = .119). Patients’ bone height after surgery compared to the bone height before surgery showed a statistically significant increase of 3.96 (67.2%) (95% CI, 3.62 to 4.30) mm, *p* < .0005.

### Complications

No post-operative complications were observed in the 35 remaining patients. All implants were osseo-integrated after 4 months of healing. At the time of prosthetic loading, an angled abutment was used in 3 out of 35 implants (8.6%) because the angle correction obtained with the N.A.B.E was insufficient for a straight abutment. All patients were observed up to 4 months post-loading.

### Angle correction assessment

The mean angular value correction from the initial osteotomy to the implant insertion ranged from 12.70 to 34.90 (mean 25.17 ± 5.10) degrees.

## Discussion

This prospective study was designed to assess the safety and effectiveness of the N.A.B.E technique in the rehabilitation of the atrophic posterior maxilla. As for all implant placements, the N.A.B.E technique requires high precision to prevent complications. The literature demonstrates lesser / reduced accuracy when implants are placed free hand versus guided (static guidance and /or dynamic navigation). Vercruyssen et al. (2014) [25] reported on a randomized, prospective study comparing the accuracy obtained when placing implants using static guidance from several providers (Materialise Universal, Facilitate) with that obtained from free-hand (“mental navigation”) and pilot-drill templates in 72 fully edentulous jaws. The mean deviations (SD) for those implants placed with static guidance were 1.4 (0.7) mm at the entry point; 1.6 (0.7) mm at the apex and 3.0 (2.0) degrees from the angular standpoint. In comparison, mean deviations (SD) measured with free-hand (“mental navigation”/ unguided) were: 2.8 (1.5) mm at entry point, 2.9 (1.5) mm at the apex and 9.9 (6.0)° for angle deviations. The above demonstrated the significant difference in deviation encountered when comparing static guidance to both the pilot guide and free hand, confirming superior accuracy with static guidance. With this kind of deviation (2.9 mm at apex and 9.9 degrees as angular error), and given the high sensitivity of the technique /method presented, attempting a free hand method could represent a high risk of sinus perforations and implant failure due to inadequate initial implant stability. Although using static guidance has been demonstrated to be significantly more accurate than free hand, using the N.A.B.E technique with static guides would require the fabrication of two guides, one for the initial osteotomy and one to direct the corrected angle preparation and implant placement, which could be very costly. Dynamic Navigation not only eliminates the need to fabricate static guides but allows for real time verification of the osteotomy and real time visualization of the adjunct anatomical structures. These are two critical advantages of the N.A.B.E technique. The accuracy of dynamic navigation is easily verified throughout the procedure by touching fixed points with the drill tip and verifying the virtual image representation with the actual patient. The accuracy of dynamic navigation has been reported in the literature. Block et al. (2017) [22] treated, in a multicentre study (3 clinicians), 100 partially edentulous patients using the X-guide dynamic navigation system and reported the following accuracy: 0.87 (0.42) mm at the coronal point, 1.56 (0.69) mm at the apex (3D) and 3.62° (2.73°) for angle deviations when the surgeries were guided. For the non-guided surgeries, the accuracy reported were 1.15 (0.59) mm as coronal deviation, 2.51 (0.86) mm as apex deviation, and 7.69° (4.92°) as angle discrepancy. A separate study by Block et al. (2017) [22] reported on the accuracy of implant placement using dynamic Navigation both fully guided and partially guided when compared to free hand. In this prospective cohort study of 478 patients with a total of 714 implants placed, Dynamic Navigation proved again to be more accurate than free hand. For fully guided navigational surgery, the mean deviation at entry point was 1.16 mm, at the apical point was 1.29 mm, and the mean angle discrepancy was 2.97 degrees. The mean deviations for implants placed free-hand/unguided were 1.78 mm at the entry point and 2.27 mm at the apical point, with a mean angle discrepancy of 6.5degrees. Their accuracy data support the conclusion that implant placement using dynamic surgical navigation is at least as good as to, if not more accurate than, static guides and substantially superior to non-guided/freehand implant surgery. Stefanelli et al. [20], reported in a retrospective observational study on 231 implants (89 arches) an error of 0.71 mm at the entry point, 1 mm at the apex, and a mean angular error of 2.26 degrees. The combined data obtained from Block et al. (2017) [24] (2017) [25] and Stefanelli et al. (2019) [20] demonstrates the increased accuracy of Dynamic Navigation when compared to both analog fabricated or fully digitally made [26] static guidance and its superior performance over free hand surgery. In this prospective study, the technique presented was done in combination with a dynamic navigation system. The residual native bone of the posterior atrophic maxilla was used to expand the bone in the ideal prosthetic position followed by the placement of implants in the absence of a bone graft. Parameters evaluated with the N.A.B.E technique included the bone gain using this technique, the implant osseointegration rate, the range of the angular value correction from the initial osteotomy to the implant insertion, the percentage of cases in which screw-retained versus cemented-retained restorations were placed and the complications occurred. The literature has reported on the same parameters evaluated using several techniques when a posterior atrophic maxilla was treated. Nedir R [27]., et al., placed 37 implants into atrophic sinuses with or without grafting for the osteotome mediated sinus elevation. The success rate reported was 91.9% (94.1% without grafting, 90.0% with graft). All implants had gained antral bone of 3.8 mm without grafting and 4.8 mm with graft. Santoro M, et al., [28] in a systematic review of the literature, reported that there were no statistically significant differences between an osteotome mediated transcrestal sinus augmentation simultaneous with implant placement whether or not bone grafting materials were used. The mean intrasinus bone gain at 3 years after surgery was 2.99 mm in cases where no grafting material was used and 4.24 mm in cases in which grafting materials were used. The mean percentage of crestal height increase at the implant site at 3 years after surgery, referring to a selection of studies with initial bone height > 4 mm, was 47.28% in procedures without grafting material and 62.68% in procedures with grafting material. A different dimensional behavior of the newly formed bone during the first 3 years after surgery was found: a slight volumetric shrinkage in grafting procedures and a slight bone increase in procedures without grafting material. No statistically significant difference in implant survival rate was found. Nedir R. et al. [29] inserted 25 implants using the osteotome sinus floor elevation technique without grafting on sites with a mean residual bone height of 5.4 mm. The reported mean sinus bone gain was 2.5 mm after the first year of follow up. In a meta- analysis evaluating the most effective method of rehabilitation of the posterior maxilla (RBH 4-8 mm) with implant-supported prostheses, Al-Moraissi EA et al. [30] analyzedthe following techniques: short implants (SI) (SIs; ≤8 mm) alone, SIs in conjunction with osteotome sinus floor elevation (OSFE) with or without bone grafting, long implants (LIs) in conjunction with OSFE with and without bone grafting, and LIs combined with lateral sinus floor elevation (LSFE) with bone grafting. They reported that moderate-quality evidence demonstrate that OSFE, combined with SI or LI placement with or without bone grafting or SI placement alone, is superior to LI placement combined with LSFE and bone grafting when used for patients with intermediate maxillary residual bone height (RBH) of 4 to 8 mm. Pjetursson BE [31] et al., reported a cumulative survival rate of 97.4% of 252 implants placed on osteotome mediated sinus augmentations on a mean 3.2 years of follow-up post-placement. According to the residual bone height, the survival rate was 91.3% for implant sites with < or = 4 mm, and 90% for sites with 4 mm and 5 mm, compared to 100% in sites with RBH bone height greater than 5 mm. According to the implant length, the survival rates were 100% for 12 mm, 98.7% for 10 mm, 98.7% for 8 mm and only 47.6% for 6 mm implants. Pesce et al. [32], evaluated the implant survival and peri-implant bone resorption around long vs normal length implants in maxillary, full-arch, immediate-loading rehabilitation of low bone quality (D4). A total of 45 patients received two mesial normal-length (10 to 15 mm) or longer (18 to 20 mm) implants and two long (18 to 20 mm) distally tilted implants. At 24 months, the use of long implants provided favorable survival and bone maintenance results in the immediate loading rehabilitation of low-quality maxillary arches. Yang J. et al. [33], in a systematic review and meta-analysis based on randomized controlled trials of maxillary sinus floor augmentations with or without grafts in the atrophic maxilla, reported no significant differences between the two groups in implant survival (*P* = 0.94), marginal bone loss (*P* = 0.73) and new bone density (*P* = 0.54). There was a significant gain in antral bone in the group that received bone grafting (*P* = 0.02). Essam Al-Moraissi [34], reported in a systematic review of the literature that there is a statistically significant correlation between Schneiderian membrane perforations and implant failure rate. In the present study, patients included in the analysis presented at the base-line with a residual bone height ranging from 4.10 to 6.90 mm (mean 5.89 ± 0.68 mm), demonstrated with linear measurements on CBCT. The bone height after the N.A.B.E ranged from 7.70 to 11.50 mm (mean 9.85 ± 1.10 mm). The mean bone gain was 3.96 mm (67.2%). The survival rate was 94.6%. When comparing the results reported in the current study, similar survival rates were encountered to those reported in the literature that employed other techniques. Using the N.A.B.E technique, 67.2% bone gain was obtained, which is consistent with transcrestal sinus augmentations in combination with bone grafting, and much greater than those reported in the literature without grafting materials. A mean angular value correction of 25.17 degrees was obtained. A total of 32 out of 35 implants received screw-retained restorations. The 3 remaining implants received cemented-retained, custom implant supported restorations due to their angles. Perforations of the sinus membrane were not encountered. Two cases were aborted due to fracture of the palatal alveolar process, which occurred while using the bone expanders for the correction of the axis. It is with no doubt that dynamic navigation is crucial in the application of the Antral Bone Expansion (N.A.B.E) technique due to the precision required in the atrophic posterior maxilla. The limitation of this study was the short observational time after the implant loading (4 months). Longer follow-up and a larger sample are needed to better understand the predictability of this technique and to draw more reliable conclusions.

## Conclusions

The introduction of Trace and Place (Trace Registration) technology in the dynamic navigation system used allows the clinician to perform a highly accurate and precise implant placement in areas with compromised anatomy, restricted access and visibility. The prospect of a total digital implant-prosthetic workflow is promising. The accuracy of the navigational technology used in this study is demonstrated by the literature. The ability to visualize in real time allows the clinician to pursue minimally invasive protocols, thus avoiding critical anatomical structures and utilizing available native bone. The N.A.B.E technique is a promising alternative for the management of the atrophic posterior maxilla, utilizing a minimally invasive approach, while avoiding bone graft surgery (the bone gain obtained using this technique (67.2%) is consistent with transcrestal sinus augmentations in combination with bone grafting). Given the short observational period post implant placement in this study, further research is necessary to evaluate the long-term survival rate.

## Data Availability

The datasets used and/or analysed during the current study are available from the corresponding author on reasonable request.

## Abbreviations

TaP: Trace and Place
CBCT: Cone beam computed tomography
CT: Computed tomography
DICOM: Digital Imaging Communication in Medicine
STL: Standard Triangulation Language
N.A.B.E: Navigated Antral Bone Expansion
mm: millimeters
°: Degree
SD: Standard deviation
SI: Short implants
OSFE: Osteotome sinus floor elevation
LSFE: Lateral sinus floor elevation
RBH: Residual bone height.
